# Prevalence and Interpersonal Correlates of Internet Gaming Disorders among Chinese Adolescents

**DOI:** 10.3390/ijerph17020579

**Published:** 2020-01-16

**Authors:** Xue Yang, Xuewen Jiang, Phoenix Kit-han Mo, Yong Cai, Le Ma, Joseph Tak-fai Lau

**Affiliations:** 1 Centre for Health Behaviours Research, Jockey Club School of Public Health and Primary Care, Faculty of Medicine, The Chinese University of Hong Kong, Hong Kong, China; phoenix.mo@cuhk.edu.hk; 2 The Chinese University of Hong Kong Shenzhen Research Institute, Shenzhen 518172, China; 3 School of Public Health, Peking University, Beijing 100191, China; jiangxuewen@pku.edu.cn; 4 School of Public Health, Faculty of Medicine, Shanghai Jiao Tong University, Shanghai 200025, China; caiyong@shsmu.edu.cn; 5 School of Public Health, Xi’an Jiao Tong University Health Science Center, Xi’an 710061, China; male@mail.xjtu.edu.cn

**Keywords:** adolescent IGD, parental factors, peers, teachers, protective factors, risk factors

## Abstract

This study investigated the prevalence and interpersonal correlates of Internet gaming disorders (IGD) among Chinese adolescents. A cross-sectional survey was conducted in two cities (Shanghai and Xi’an) in China. A total of 2666 (Mean_age_ = 12.77 ± 0.75) year-one students from eight middle schools completed a self-reported questionnaire. It tested their levels of IGD, parental psychological control, negative interpersonal events (physical/verbal abuse by parents, verbal abuse by teachers, peer/online bullying), social support from parents/peers, and positive relationships with parents/peers. Results showed that 346 participants (13.0%) were classified as having IGD. Gender, city, single-parent family, family socio-economic status, and mother’s education level were significantly associated with the risk of IGD. Logistic regression analyses with and without controlling for the significant background variables showed that the studied interpersonal variables were significantly associated with IGD, respectively. Forward stepwise logistic regression showed that the significant correlates of IGD included parental psychological control, physical/verbal abuse by parents, verbal abuse by teachers, and peer/online bullying. Results highlight the importance of addressing interpersonal risk factors to reduce adolescent IGD. Limitations and implications of this study are discussed.

## 1. Introduction

Internet gaming disorder (IGD) is a newly defined disease. It was included as a condition for further study in the latest (fifth) edition of the Diagnostic and Statistical Manual of Mental Disorders (DSM-5) [1]. Furthermore, gaming disorder, which includes both online and offline gaming, was recently included as a formal diagnostic entity in the 11th edition of the International Classification of Diseases (ICD-11) [2], and distinguished from hazardous gaming. Prevalence of adolescent IGD varies across studies, due to differences in regions, assessment criteria, and socio-cultural variables involved in the studies [3]. A systematic review reported that it ranged from 3.5% to 17% in mainland China [4]. In Taiwan, 46% of the regular adolescent Internet game players had had online game addiction as measured by the Online Games Addiction Scale [5]. Two studies conducted in Hong Kong that used the Game Addiction Scale reported prevalence of gaming addiction (included both online and video gaming) of 15.6% (N = 503) [6] and 13.0% (N = 920) [7], respectively.

There are several types of potential protective and risk factors of IGD, including contextual factors (e.g., convenience and accessibility), interpersonal factors (e.g., interpersonal conflict and social support), personal factors (e.g., biological factors, personality traits, mental/emotional problems, and self-esteem, loneliness), and game-related factors (e.g., game genres, instant gratification, interactivity, and anonymity nature of online gaming) [8,9,10]. Some researchers pointed out that interpersonal factors of IGD have been under-researched [4,11,12]. Interpersonal factors have been highlighted as a crucial role in understanding and addressing internal and external behavioral problems of adolescents [13,14]. This study focused on potentially important interpersonal correlates of adolescent IGD. Such factors will help to guide the development of interpersonal/family-based interventions.

Family and parents play central roles in adolescents’ socialization, behavioral development, and health [15]. Increasing studies have identified various family-related risk factors of IGD, including parental modelling of online gaming (e.g., parental gaming behavior [16] and parental attitudes [11]), poor parent-child relationship (e.g., perceived parental inconsistency [17], parental rejection [8], poor parental supervision [11,18]), poor family environment (e.g., poor family functioning [11], perceived family disharmony [6], and poor family cohesion [19]). Traditional Chinese culture and some Asian cultures emphasize parental psychological control (i.e., parental control that intrudes on the psychological and emotional development of the child) [20]; the attribute is a stressor and an interpersonal risk factor of adolescents’ mental distress and behavioral problems [21,22,23,24,25]. A dearth of research has investigated the roles of parental psychological control and physical/verbal abuse by parents in development of IGD among adolescents. Furthermore, some family-related factors are protective against IGD (e.g., positive parent-child relationship [26] and parental support [11]). Such risk and protective factors were tested in this study.

In addition, school teachers may affect adolescent IGD. A longitudinal study has shown that teachers’ autonomy support could reduce adolescent IGD [27]. Tension may exist in the teacher-student relationship, as Chinese culture emphasizes obedience and high teachers’ expectation on students’ academic performance is common. Verbal abuse exerted by teachers is another potential risk factor of adolescent IGD. To our knowledge, no study has investigated such associations.

Peer influences are also important determinants of adolescent health-risk behaviors. Previous studies have revealed that relationships with peers (e.g., deviant peer affiliation, number of gamers among friends’ networks, and peer support) were significantly associated with IGD [4]. According to the report of UNESCO [28], one-third of the children and adolescents worldwide had been bullied, while 5%–21% of the children and adolescents had been experienced cyberbullying. Previous studies found that peer bullying was significantly associated with IGD among college students [29] and pathological Internet use among adolescents [30]. Online bullying has also been anecdotally and empirically linked to multiple maladaptive emotional and behavioral outcomes among adolescents, such as depression, substance use, and suicide [31,32]. Yet, our literature search did not locate any studies that examined the association between online/peer bullying and adolescent IGD. Good peer relationships, such as high quality peer relationship and peer support, can be protective against adolescent IGD [4]; the associations were tested in this study.

The present study tested the significance of some under-examined inter-personal risk factors of IGD among Chinese adolescents, including those related to parents (perceived parental psychological control, physical/verbal abuse by parents), teachers (verbal abuse by teachers), and peers (online bullying and peer bullying). In addition, interpersonal protective factors of IGD (social support obtained from parents and peers, and positive relationships with parents and peers) were also investigated. It is hypothesized that perceived parental psychological control, physical/verbal abuse by parents, verbal abuse by teachers, online bullying and peer bullying would be positively associated with IGD, while social support obtained from parents and peers and positive relationships with parents and peers would be negatively associated with IGD.

## 2. Methods

### 2.1. Study Design and Participants

This cross-sectional survey was conducted in two large Chinese cities (i.e., 1423 Shanghai in Eastern China and 1243 Xi’an in Western China), that have population sizes of 23,019,148 and 9,056,800, respectively. All year-one students of eight conveniently selected middle schools (five in Shanghai and three in Xi’an) were invited to participate in this study. They completed a self-reported questionnaire in classroom settings in the absence of teachers, and being supported by a research assistant. It took about 20 min to complete the questionnaire. Trained and experienced research assistants briefed the participants about the study and answered questions related to the questionnaire. Consent was sought from the school principals before the survey. Students were assured that participation is voluntary and refusal or quitting the survey has no negative consequences; it was announced that return of the completed questionnaire implied informed consent. No incentive was provided to the participants. The study was approved by The Survey and Behavioral Research Ethics Committee of the corresponding author’s university (Ref# 055-18).

### 2.2. Measures

IGD was measured by the 9-item DSM-5 IGD symptoms checklist. It is a short, user-friendly, self-report measure assessing DSM-5 IGD symptoms [1] that include preoccupation, tolerance, withdrawal, unsuccessful attempts to limit gaming, deception or lies about gaming, loss of interest in other activities, use despite knowledge of harm, use for escape or relief of negative mood, and harm in the past 12 months. Response options include no (0) and yes (1). A score of 5 is taken as the cutoff point for defining IGD. The Chinese version was found to have good psychometric properties [33,34]. It has been applied in a number of studies [33,34,35]. The scale reliability was acceptable in the current study (Cronbach’s alpha = 0.78).

Parental psychological control was measured by the 8-item Psychological Control Scale—Youth Self-Report [36]. This scale taps three major psychologically controlling tactics, i.e., guilt induction (e.g., “My mother/father blames me for other family members’ problems”), invalidation of feelings (e.g., “My mother/father is always trying to change how I feel or think about things”), and love withdrawal (e.g., “My mother/father will avoid looking at me when I have disappointed her/him”) (1 = never to 4 = always). The Chinese version has been used in a previous study [37]. The scale reliability was good in the current study (Cronbach’s alpha = 0.82).

Negative interpersonal events/experiences with parents, teachers, and peers were measured by four questions: (1) Physical or verbal abuse by parents was measured the item: “During the last year, how often had you been scolded, criticized or physically abused by your parents?” [38] (2) Verbal abuse exerted by teachers: “During the last year, how often had you been scolded or criticized by your school teachers?” [38] (3) Peer bullying: “During the last year, how often have you been threatened, bullied, or abused by peers?” (4) Online bullying: “During the last year, how often have you been threatened, bullied, or abused online?” The item responses ranged from 0 (never) to 4 (always). These questions were created by a research panel that included health psychologists and epidemiologists worked in the area of adolescent mental health research.

Perceived support obtained from parents and peers was each measured by two questions, respectively [39]. The two items were “How much support had you received from your parents when you needed to talk with someone or needed emotional support?” and “How much support had you received from your family when you needed instrumental support (e.g., financial support)?” The items are rated on 7-point Likert Scales ranged from 1 (none) to 7 (tremendous). To assess peers’ support, the wording “parents” were replaced by “peers”. Good reliability was obtained for the family’s (Cronbach’s alpha = 0.91) and peer’s (Cronbach’s alpha = 0.93) scales, respectively.

Two single item scales were created to assess qualities of the relationships with parents and peers. In the two items, participants were asked to rate their relationships with their parents and peers, respectively. The response scores ranged from 1 (very poor) to 10 (very good).

#### Statistical Analysis

Descriptions of the background variables and interpersonal variables of the participants are presented. Chi-square test was conducted to test the associations participants’ background characteristics and IGD. Univariate logistic regression was used to analyze the associations between each of the interpersonal variables and IGD; univariate odds ratios were reported (ORu; model_u_). Adjusted odds ratios (ORa) were obtained by fitting logistic regression models for each of the studied factors in single-variable logistic regression models (Model_a_) that adjusted for the background variables that were significantly associated with IGD. A summary forward stepwise logistic model was also fit and its odds ratios (ORstep) were reported. We did not enter all independent variables into a single model because of high correlations (collinearity) among these variables. All odds ratios were accompanied by their respective 95% confidence intervals (CI). SPSS version 18.0. was used for data analysis. The level of statistical significance was set at 0.05.

## 3. Results

### 3.1. Background Characteristics

A total of 2666 middle school students participated in the study. Their mean age (SD) was 12.77 ± 0.75 years. As showed in Table 1, 51.9% were male; 84.5% lived with both parents (9.7% lived in single-parent family); 58.1% self-reported good or very good family socio-economic status. About half of the participants reported that their father (*n* = 1495; 56.1%) and mother (*n* = 1472; 55.2%) had attained a college or higher education level. The mean score of parental psychological control (2.51) was closed to its midpoint (2.50). Some mean scores were slightly higher than the midpoints (parental/peer support = 5.16/5.09 versus midpoint = 4; quality of relationships with parents/peers = 8.04/9.31 versus midpoint = 5.50). The mean scores of the negative interpersonal experiences were lower than the midpoint of 2.50 (physical/verbal abuse by parents = 1.34, verbal abuse by teachers = 1.60, peer bullying = 0.95, online bullying = 0.35; Table 1); 17.6%, 12.7%, 8.2%, and 2.8% of the participants endorsed that they had “often” or “always” experienced physical/verbal abuse exerted by parents, verbal abuse exerted by teachers, peer bullying, and online bullying, respectively.

### 3.2. Prevalence of IGD

A total of 346 participants (13.0%; 95% CI: 11.7%–14.3%) were classified as having IGD. Male students showed higher prevalence than female students (17.5% versus 7.9%; *p* < 0.001). Xi’an reported higher prevalence than Shanghai (14.4% versus 11.7%; *p* < 0.001). Adolescents who lived in single-parent families had significantly higher prevalence of IGD (23.6% versus 11.8%; *p* < 0.001) than their counterparts. Moreover, mothers’ education level was significantly associated with IGD (*p* = 0.01; Table 2). Post hoc tests showed that the participants whose mothers had had primary school or below education were more likely to have IGD than those whose mothers had had high school/vocational school (*p* = 0.05), college (*p* = 0.01), or undergraduate education (*p* = 0.03). The participants whose mothers had had middle school education were more likely to have IGD than those whose mothers had had college (*p* = 0.004), or undergraduate education (*p* = 0.03). These results are not tabulated.

### 3.3. Logistic Regression Analysis

As showed in Table 3, univariate logistic regression showed that the studied interpersonal variables were significantly associated with IGD, respectively (*p* < 0.001). Similar results were obtained when adjusted for the significant background factors of IGD (gender, city, single-parent family, and mother’s education level). Specifically, significant family-related risk factors of IGD included parental psychological control (ORa = 1.82) and parental verbal/physical abuse (ORa = 1.59); protective family-related factors included perceived parental support (ORa = 0.78) and positive relationship with parents (ORa = 0.84). Significant teacher-related risk factors included verbal abuse by teachers (ORa = 1.63). Significant peer-related risk factors included peer bullying (ORa = 1.85) and online bullying (ORa = 1.76); significant protective factors included perceived peer support (ORa = 0.78) and perceived positive relationship with peers (ORa = 0.84).

In the forward stepwise model that considered all the studied independent variables for entry, the five significant risk factors were parental psychological control (ORstep = 1.34), parental verbal/physical abuse (ORstep = 1.16), verbal abuse by teachers (ORstep = 1.13), peer bullying (ORstep = 1.11), and online bullying (ORstep = 1.34).

## 4. Discussion

Adolescents are at high-risk of developing IGD [40]. Our study found prevalence of IGD of 13%, which is the same (13%) as that reported in Wang’s study conducted among Chinese adolescents in Hong Kong and based on another instrument [7]. We found that male adolescents were much more likely to have IGD than female adolescents (17.5% versus 7.9%); the observed significant sex difference is consistent with that reported in previous studies (e.g., [4]). In this study, disadvantaged family background (e.g., single-parent family, lower socio-economic status (SES), and lower maternal education level) were found to be significant risk factors of IGD, corroborating previous studies (e.g., [8]). SES is in general negatively associated with both risk behaviors and risk factors of such risk behaviors [8]. Attention is hence needed to strengthen prevention of IGD among disadvantaged adolescents. It is also important for future studies to look at whether SES would potentially moderate the associations between sex and other risk factors and IGD. Furthermore, adolescents in Xi’an were more likely to have IGD than those in Shanghai, although Shanghai is a developed mega-city. The difference reminds us that the prevalence of IGD may vary across regions of a country, especially in a vast country like China.

We tested both positive (i.e., social support and positive interpersonal relationships) and negative (i.e., perceived parental psychological control and negative interpersonal events/experiences) interpersonal correlates of adolescent IGD and the results support our hypotheses. Consistent with previous studies [11,18], we found that perceived support obtained from parents and peers and perceived positive interpersonal relationships with parents and peers were significantly associated with IGD. It is known that perceived social support and positive relationships with parents/peers are important resources of resilience and positive coping, as previous studies have shown that such resources could reduce stress, facilitate adaptive coping, and prevent maladaptive behaviors among adolescents [12]. Thus, students being supported by parents and teachers would be less likely than others to engage in negative stress coping; escapism was associated with IGD [41]. Furthermore, future studies may look at the interactions between various protective factors of IGD (positive and supportive interpersonal relationships), as well as interactions between protective factors of IGD involving parents and teachers. Furthermore, effective interventions for reduction of IGD can be strengthened by cultivation of positive family and school environments.

We have identified a number of significant risk correlates of adolescent IGD, including those related to parents (parental psychological control and parental verbal/physical abuse), teachers (verbal abuse by teachers), and peers (online bullying, and peer bullying). We believe that this is the first study that showed parental psychological control may increase adolescent IGD. Consistently, previous studies have demonstrated that this parental psychological control, as a form of intrusive parental behavior, was linked to various forms of internalized adolescent problems (e.g., depression, loneliness, withdrawal, eating disorders) [21,22,23,24,25]. Parents are primary caregivers of adolescents; parental abuse induces strong distress and risk behaviors among adolescents [42]. In this sample, about one in five (17.6%) of the adolescents reported that they often or always experienced parental verbal/physical abuse in the last year. Parental verbal and physical abuse may be more prevalent in Chinese versus Western families [43]. In this study, physical/verbal abuse exerted by parents was significantly associated with adolescent IGD. The finding is consistent with a previous study finding that childhood trauma experience, including physical and verbal abuse, may increase the risk of IGD, and children’s stress vulnerability may be a mediator [44].

Readers may interpret the findings in a cultural context. Both parental psychological control and physical/verbal abuse are potential manifestations of the Chinese and Asian cultures that emphasize obedience to parental authority and strong control over children and adolescents. A demonstration is the Chinese saying that: “A father is at fault if he was not harsh; a teacher is lazy if he was not strict”. In contrast, adolescents are experimenting with the quest for autonomy and tend to be ‘rebellious’ [20]. The negative parenting practices of strong parental control (both psychological and behavioral) over the adolescents may impede the increasing need of autonomy demanded by adolescents and cause severe parent-adolescent conflicts; the resulting heavy strains may induce maladaptive coping such as excessive Internet gaming among adolescents [20]. The finding hence suggests that the Chinese culture that emphasizes obedience and authoritarian parental style might contribute to the higher prevalence of IGD in China and Asia, compare to some Western countries. Future research is warranted to test this hypothesis. The findings also suggest that intervention programs for adolescent IGD may consider promoting positive parenting styles and non-violent communication skills among parents of adolescents. Interventions involving parents may increase the effectiveness of IGD treatment.

Following the same vain concerning parental psychological control and abuse, teachers’ verbal abuses might also be more common in Asian than Western countries. In this sample, about one in seven (12.7%) of the participants reported that they had often or always been verbally abused by their teachers in the last year. Supporting the literature that teachers greatly influence adolescent behavioral health and development [27], we found a significant positive association between teachers’ verbal abuse and IGD. A dearth of research has investigated teachers’ role in adolescent IGD. One of the several studies suggests that teachers played a crucial role in students’ satisfaction of basic psychological needs (e.g., relatedness and autonomy); deprivation of such needs reduced adolescent IGD [27,45]. Accordingly, it is plausible that teachers’ verbal abuses had reduced adolescents’ satisfaction of their basic psychological needs (e.g., relatedness and autonomy), and increased the risk of IGD. Furthermore, challenges to self-esteem and self-confidence and peer rejection may be other plausible explanations of the relationship between teachers’ verbal abuse and adolescent IGD, as verbal abuse is likely to occur in front of other classmates. Such attributes are associated with IGD [8,9,10]. Future studies may test these possible mechanisms. More studies are warranted to understand the role of teachers’ communication style and IGD (and other risk behaviors) among adolescents. Effective interventions for reduction of IGD should thus involve teachers, whom should be made aware of the potential harms of their verbal abuse, including increasing IGD. Training teachers on positive communication skills is potentially important. As the school environment is an important part of the socio-ecological model of health, structural interventions to modify teachers’ culture and norms are warranted.

Furthermore, this study highlights the potential significance of bullying in risk of IGD among adolescents. We found positive associations between peer/online bullying and adolescent IGD. Bullying is certainly a significant negative experience; it may induce loneliness, social anxiety and perceived stress and reduce self-esteem and social competence [46]; it is hence a prominent risk factor of many severe mental and behavioral problems including depression and suicide [46]. As loneliness, social anxiety, perceived stress, and depression are well-documented factors of IGD, such distresses caused by bullying may explain how bullying has increased IGD. Such mediation hypotheses should be tested in future work. With the sharp increase in smartphone and computer use, various forms of online bullying may have become a common experience among adolescents [47,48]. Conceptually, peer bullying may overlap with online bullying, as the same person may bully another person both online and offline. However, both the variables of peer and online bullying were significant in the final stepwise model, suggesting that both online and offline bullying might increase the risk of IGD independently. Future study should refine the measurements of bullying occurring online and offline, and test their independent and/or interactive effects on IGD. The finding extends the spectrum of harms potentially caused by peer and online bullying to the risk of IGD. Furthermore, although our sample reported relatively low prevalence of peer/online bullying, given its various negative consequences, it is important to reduce online bullying and empower bullied victims to adaptively cope with the problem and prevent escapism to Internet gaming.

The study has some limitations. First, the cross-sectional design could not address the causal effects of these interpersonal factors on adolescent IGD. It is plausible that IGD may increase interpersonal conflicts and strains, or the relationship between IGD and these interpersonal correlates may be reciprocal. Some interpersonal-level factors, such as poor parental competence and father-mother relationships, are more likely to have causal effects on adolescent IGD and need to be investigated in future work. It is important to continually monitor changes in prevalence of IGD over time alongside with changes in interpersonal factors to observe possible effects of each other in cross-lagged analyses. Second, the schools were conveniently selected and may not be representative; the geographic limitation may also prevent national generalization. Caution is needed when generalizing the findings to other cultures. Third, all the scores were self-reported perceptions; we did not collect information from adolescents’ teachers, parents, and peers as it is not too feasible; dyadic studies would, however, be more informative. Fourth, IGD was measured by self-reported DSM-5 items, and did not reflect the recent change in the ICD-11 classification. Presently, the tools for ICD-11 classification are still under development. Future studies that look at clinical diagnosis of IGD would be informative. Electronic measurement of Internet gaming may offer a more objective measure to investigate Internet gaming behaviors (e.g., time use of Internet gaming). We suggest that future studies can include such measures to better understand Internet gaming behaviors among adolescents. Fifth, this study only included general interpersonal factors instead of interpersonal variables that are specifically related to IGD or Internet gaming/use (e.g., parental control over IGD). Future studies are suggested to consider such factors. Lastly, some of the variables (e.g., peer/online bullying) were assessed by the single-item scales. The results should be confirmed by using the validated scales in future work.

## 5. Conclusions

The present study represents one of the first attempts to identify a wide range of significant interpersonal risk and protective correlates of adolescent IGD. The strain caused by difficult interpersonal relationships with parents, teachers, and peers is an under-emphasized dimension among the determinants of adolescent IGD. The findings are consistent with those of previous empirical studies, that interpersonal stressors could in general significantly increase adolescent risk behaviors. Some novel factors (e.g., parental psychological control) of IGD have also been identified. In contrast, support and quality relationships with parents and peers were protective against adolescent IGD, and should be cultivated. The relationships between interpersonal correlates and IGD should be interpreted cautiously, since the data did not allow claims of a causal effect, nor can it be generalized to other populations or other cultures. It is greatly important for future studies to look at the interactions between interpersonal risks and protective factors, and test efficacy of intervention programs for IGD reduction that take the studied interpersonal risk and protective factors into account. The interventions that aim to teach interpersonal communication skills to both adolescents and their important others (e.g., families, peers, teachers) and adaptive strategies to cope with interpersonal stress to help to cultivate supportive family and school environment and reduce interpersonal conflicts may be effective to prevent and reduce IGD.

## Figures and Tables

**Table 1:** Background characteristics the participants and mean scores of the psychosocial scales (N = 2666).

Background/Psychosocial Variables	Mean ± SD	*n* (%)
Age	12.77 ± 0.75	
Gender (male)		1384 (51.9)
City (Shanghai)		1423 (53.4)
Live with		
Father		83 (3.1)
Mother		219 (8.2)
Both		2252 (84.5)
Neither		112 (4.2)
Single-parent family (no)		2407 (9.3)
Family socio-economic status		
Very poor		16 (0.6)
Poor		93 (3.5)
Ordinary		1008 (37.8)
Good		1333 (5.0)
Very good		216 (8.1)
Education level (father)		
Primary school or below		75 (2.8)
Junior high school		459 (17.2)
Senior high school/vocational school		637 (23.9)
College		360 (13.5)
Undergraduate		834 (31.3)
Postgraduate		301 (11.3)
Education level (mother)		
Primary school or below		112 (4.2)
Junior high school		469 (17.6)
Senior high school/vocational school		613 (23.0)
College		416 (15.6)
Undergraduate		848 (31.8)
Postgraduate		208 (7.8)
IGD (yes)		346 (13.0)
Parental support (score ranging 1–7)	5.16 ± 1.43	
Peer support (score ranging 1–7)	5.09 ± 1.33	
Positive relationship with parents (score ranging 1–10)	8.04 ± 2.04	
Positive relationship with peers (score ranging 1–10)	8.31 ± 1.84	
Parental psychological control (score ranging 1–4)	2.51 ± 0.66	
Verbal abuse by teachers (score ranging 0–4)	1.60 ± 0.90	
Verbal/physical abuse by parents (score ranging 0–4)	1.34 ± 0.97	
Peer bullying (score ranging 0–4)	0.95 ± 0.86	
Online bullying (score ranging 0–4)	0.35 ± 0.77	

**Table 2:** The prevalence of Internet gaming disorder (IGD) and its Chi-square test in adolescents with different characteristics.

Characteristics	*n* (%)	Value	*p* Value
Gender		53.45	<0.001
Male	242 (17.5)		
Female	101 (7.9)		
City		4.17	0.04
Shanghai	167 (11.7)		
Xi’an	179 (14.4)		
Single-parent family		28.03	<0.001
Yes	61 (23.6)		
No	284 (11.8)		
Family socio-economic status		32.96	<0.001
Very poor	9 (56.3)		
Poor	19 (2.4)		
Ordinary	133 (13.2)		
Good	151 (11.3)		
Very good	24 (11.1)		
Education level (father)		8.08	0.15
Primary school or below	11 (14.7)		
Junior high school	73 (15.9)		
Senior high school/vocational school	85 (13.3)		
College	45 (12.5)		
Undergraduate	88 (1.6)		
Postgraduate	41 (13.6)		
Education level (mother)		16.48	0.01
Primary school or below	22 (19.6)		
Junior high school	76 (16.2)		
Senior high school/vocational school	77 (12.6)		
College	38 (9.1)		
Undergraduate	98 (11.6)		
Postgraduate	32 (15.4)		

**Table 3:** Logistic regression analyses of interpersonal variables of IGD.

Variables	Model_u_			Model_a_			Model_step_		
	ORu	95% CI	*p* Value	ORa	95% CI	*p* Value	ORstep	95% CI	*p* Value
Parental support	0.78	0.72–0.84	<0.001	0.78	0.72–0.86	<0.001			
Peer support	0.77	0.71–0.84	<0.001	0.78	0.71–0.86	<0.001			
Positive relationship with parents	0.85	0.80–0.89	<0.001	0.84	0.79–0.89	<0.001			
Positive relationship with peers	0.85	0.80–0.90	<0.001	0.84	0.79–0.90	<0.001			
Parental psychological control	1.84	1.55–2.19	<0.001	1.82	1.48–2.23	<0.001	1.34	1.07–1.68	0.01
Verbal abuse by teachers	1.79	1.58–2.02	<0.001	1.63	1.41–1.89	<0.001	1.13	0.96–1.33	0.14
Verbal/physical abuse by parents	1.68	1.50–1.87	<0.001	1.59	1.40–1.81	<0.001	1.16	1.00–1.34	0.05
Peer bullying	1.96	1.74–2.20	<0.001	1.85	1.61–2.12	<0.001	1.11	0.96–1.27	0.15
Online bullying	1.75	1.56–1.96	<0.001	1.76	1.53–2.03	<0.001	1.34	1.16–1.56	<0.001

Note: Model_u_: univariate logistic regression for each interpersonal variable; Model_a_: logistic regression with adjusted for the significant background variables; Model_step_: forward stepwise logistic regression with adjusted for the significant background variables; 95% CI = 95% confidence intervals.
